# Comparative analysis of SmartArc‐based dual arc volumetric‐modulated arc radiotherapy (VMAT) versus intensity‐modulated radiotherapy (IMRT) for nasopharyngeal carcinoma

**DOI:** 10.1120/jacmp.v12i4.3587

**Published:** 2011-11-15

**Authors:** Tsair‐Fwu Lee, Pei‐Ju Chao, Hui‐Min Ting, Su‐Hua Lo, Yu‐Wen Wang, Chiu‐Ching Tuan, Fu‐Min Fang, Te‐Jen Su

**Affiliations:** ^1^ Medical Physics & Informatics Lab., Department of Electronics Engineering National Kaohsiung University of Applied Sciences Kaohsiung; ^2^ Department of Radiation Oncology Kaohsiung Chang Gung Memorial Hospital and Chang Gung University College of Medicine Kaohsiung; ^3^ Department of Radiation Oncology Chi Mei Medical Centre Liouying Tainan; ^4^ Department of Electronics Engineering National Taipei University of Technology Taipei Taiwan

**Keywords:** dual arc, VMAT, IMRT, NPC

## Abstract

The purpose of this study was to evaluate and quantify the planning performance of SmartArc‐based volumetric‐modulated arc radiotherapy (VMAT) versus fixed‐beam intensity‐modulated radiotherapy (IMRT) for nasopharyngeal carcinoma (NPC) using a sequential mode treatment plan. The plan quality and performance of dual arc‐VMAT (DA‐VMAT) using the ${\text{Pinnacle}}^{3}$ Smart‐Arc system (clinical version 9.0; Philips, Fitchburg, WI, USA) were evaluated and compared with those of seven‐field (7F)‐IMRT in 18 consecutive NPC patients. Analysis parameters included the conformity index (CI) and homogeneity index (HI) for the planning target volume (PTV), maximum and mean dose, normal tissue complication probability (NTCP) for the specified organs at risk (OARs), and comprehensive quality index (CQI) for an overall evaluation in the 11 OARs. Treatment delivery time, monitor units per fraction (MU/fr), and gamma $\left(\Gamma_{3\text{mm},3\%}\right)$ evaluations were also analyzed. DA‐VMAT achieved similar target coverage and slightly better homogeneity than conventional 7F‐IMRT with a similar CI and HI. NTCP values were only significantly lower in the left parotid gland (for xerostomia) for DA‐VMAT plans. The mean value of CQI at 0.98±0.02 indicated a 2% benefit in sparing OARs by DA‐VMAT. The MU/fr used and average delivery times appeared to show improved efficiencies in DA‐VMAT. Each technique demonstrated high accuracy in dose delivery in terms of a high‐quality assurance (QA) passing rate (>98%) of the $\left(\Gamma_{3\text{mm},3\%}\right)$ criterion. The major difference between DA‐VMAT and 7F‐IMRT using a sequential mode for treating NPC cases appears to be improved efficiency, resulting in a faster delivery time and the use of fewer MU/fr.

PACS number: 87.53.Tf, 87.55.x, 87.55.D, 87.55.dk

## I. INTRODUCTION

Nasopharyngeal carcinoma (NPC) is highly sensitive to ionizing radiation, and radiation therapy (RT) is the mainstay treatment modality for nonmetastatic disease. Various methods have been used to improve local control by increasing the target coverage, including three‐dimensional conformal radiotherapy (3D CRT)^(^ ^1^ ^)^ and intensity‐modulated radiotherapy (IMRT).^(^ ^2^ ^,^ ^3^ ^)^ IMRT enables the simultaneous delivery of different doses to different target volumes, representing an ideal technique for localized dose escalation.^(^ ^4^ ^)^ Volumetric‐modulated arc therapy (VMAT), which makes various gantry arcs with dynamic multileaf collimation to modulate the fluence map, has also been used.^(^ ^5^ ^–^ ^7^ ^)^ With the capability of delivering a highly conformal dose distribution within a short time interval, VMAT has been widely accepted by the radiotherapy community.

VMAT plans performed by the RapidArc algorithm in the Eclipse (Varian Medical Systems, Palo Alto, CA, USA) treatment planning system (TPS) have been reported to have faster delivery times, use fewer monitor units (MU), and have superior dose distributions than conventional TPS with IMRT.^(^ ^8^ ^–^ ^13^ ^)^ The novel TPS SmartArc, developed by Philips Radiation Oncology Systems (Philips, Fitchburg, WI, USA) in collaboration with RaySearch Laboratories AB (Stockholm, Sweden) and which builds on the existing direct machine parameter optimization (DMPO)^(^ ^14^ ^)^ functionality in ${\text{Pinnacle}}^{3}$, has recently become clinically and commercially available and can generate VMAT planning for both Elekta (Elekta Ltd., Crawley, UK) and Varian accelerators.^(^ ^15^ ^–^ ^17^ ^)^ Another RaySearch‐based and linear accelerator (linac) vendor‐independent TPS, Oncentra MasterPlan v.3.3 (Nucletron BV, Veenendaal, The Netherlands), became clinically available in December 2009 and permits VMAT optimization for both linacs. The underlying VMAT planning engine in both SmartArc and Oncentra MasterPlan TPS is the same as that developed by RaySearch, and the dose calculation algorithms of pencil beam (PB) convolution and collapsed cone convolution (CCC) were chosen.^(^ ^18^ ^,^ ^19^ ^)^

Several reports have demonstrated the dosimetric superiority or similarity of VMAT applied in head and neck (HN) patients.^(^ ^12^ ^,^ ^13^ ^,^ ^15^ ^,^ ^20^ ^,^ ^21^ ^)^ However, reported studies comparing VMAT and IMRT in NPC are rare. In the present study, we sought to evaluate and quantify the planning performance of SmartArc‐based VMAT, and to compare the dose distribution with existing conventional IMRT techniques.

## II. MATERIALS AND METHODS

### A. Study population and conditions

Eighteen NPC patients curatively treated using a sequential step‐and‐shoot 7F‐IMRT technique, planned originally with ${\text{Pinnacle}}^{3}$ (ver. 7.4; Philips), were examined. The median age was 48 (range, 35–68) years, with 12 males and six females. The clinical stage distribution according to the American Joint Committee on Cancer (AJCC 6th edition) staging system was I–II in nine (50%) patients, III in seven (39%), and IVa–b in two (11%). Fourteen (78%) patients were treated with a combination of chemotherapy. The present study was approved by the institutional review boards of the hospitals involved (IRB 99–1420B).

### B. Linac and the record and verify system

The delivery chain studied in this work included an Elekta linac and a MOSAIQ (v. 1.60Q3) record and verify system (R&V; IMPAC Medical Systems, Inc., Sunnyvale, CA, USA). Treatment plans were transferred via Digital Imaging and Communications in Medicine (DICOM) RT from the TPS to the MOSAIQ.

All IMRT and VMAT plans were created using the same 6 MV photon beams commissioned for an Elekta Synergy linac equipped with an 80‐leaf 1 cm multileaf collimator (MLC) (40 leaf pairs, maximal leaf speed of 2.0 cm/s and both static/dynamic minimum leaf gaps were 0.50 cm, the minimum MU/cm leaf travel was 0.30 MU/cm, total leaf travel distance was 32.5 cm, and the leaves did not interdigitate), upper jaws, and backup jaws covering a full $40\times 40\;{\text{cm}}^{2}$ field (maximal jaw speed of 1.5 cm/s), maximal gantry speed of 6°/s (the minimum and maximum MU/° of gantry rotation were 0.10 MU/° and 20.0 MU/°, respectively), and variable dose rate up to 500 MU/s (seven fixed dose rate levels were available, each half the dose rate of the next higher level without continuous adjustment). Actual dose rates differed from nominal dose rates by ±25%. The combination of the dose rate, gantry speed, and leaf speed was automatically selected optimally by the linac control system Precise Desktop 7.01 during VMAT delivery.^(^ ^21^ ^–^ ^23^ ^)^

### C. Treatment planning system

Treatment planning was performed and replanned with the ${\text{Pinnacle}}^{3}$ SmartArc (version 9.0); the hardware used for the TPS was the Philips 810X (64‐bit) Computer Workstation (CPU type: Intel Xeon Quad‐Core 2.8 GHz) with 16 GB RAM, and the operating system used was the Sun Solaris 10 x64. This version supports VMAT planning for Elekta treatment units with single arc, dual arc, or multiple individual arcs.

The SmartArc treatment planning module has been discussed in several reports.^(^ ^16^ ^,^ ^17^ ^,^ ^24^ ^,^ ^25^ ^)^ Briefly, the initial arc parameters including arc length, delivery time, and number of arcs were determined. SmartArc performs inverse planning with control points (CPs), beginning with the first CP of the first beam. Coarse segments around the arc are generated with an equal separation of 24°. Intensity‐modulation optimization is performed on the fluence maps of each of these segments. A PB algorithm was employed based on the singular value decomposition (SVD) approach proposed by Bortfeld et al.^(^ ^26^ ^)^ The fluence maps are converted to MLC segments for each discrete beam orientation using the sliding‐window conversion algorithm. To satisfy the static machine constraints, 2–4 leaf and jaw segments were generated for each map, depending on the degree of modulation. After MLC segment filtering, only two leaf and jaw segments remained with the highest number of open‐leaf pairs and met the mechanical constraints of the particular MLC (e.g., the avoidance of interdigitation). The segments were redistributed around the arc length, and linearly interpolated segments were added to reach a final fine arc spacing. The resulting segments were optimized using machine‐parameter optimization to satisfy dose–volume objectives (DVOs), which were constrained by the leaf motion, dose rate, and gantry speed. The machine parameters were optimized using an iterative, nonrandomized gradient‐based optimization algorithm after the MLC and jaw segments were created. Finally, convolution dose calculation using a CCC algorithm was performed to recover potential errors from the PB dose calculation used during optimization. Segment‐weight optimization was also performed on the final segments. This result is a single dynamic arc beam that is deliverable within the linac's machine‐commission parameters.^(^ ^5^ ^–^ ^7^ ^,^ ^13^ ^,^ ^17^ ^,^ ^27^ ^)^ SmartArc planning steps are summarized as follows:

1. Add a dynamic arc beam.
2. Set the couch, collimator, and beam angles.
3. Set the DVOs.
4. Specify SmartArc optimization parameters.
5. Optimize.
6. Compute final convolution dose.

### D. Planning objectives

Patients were immobilized from head to shoulders using commercially available thermoplastic masks and an individually customized bite block. Computed tomography (CT) images (2.5 mm slice thickness) acquired from the top of the vertex to the level of the carina, taken from a CT simulator (LightSpeed RT16; GE Medical Systems, Milwaukee, WI, USA) containing 512 × 512 pixels in each slice, with the dimensions of $0.9375\times 0.9375\times 2.5\;{\text{mm}}^{3}$, were used. The field of view had a mean dimension of 48 cm. The gross tumor volume (GTV) covered the visible primary tumor and neck nodes >1 cm in diameter or nodes with necrotic centers. The clinical target volume (CTV) encompassed the GTV with at least a 1.5 cm margin, microscopic spread of disease, and prophylactic neck area. The planning target volume (PTV) included the CTV with 3 mm extensions in all dimensions to account for patient setup error and motion uncertainties; the PTV ranged from 533.6 to 745.9 cc (655.8±295.5). The prescribed dose/fractionation was 72 Gy to the ${\mathrm{PTV}}_{72}$, 59.4 Gy to the elective ${\text{PTV}}_{59.4}$, and 50.4 Gy $\left({\text{PTV}}_{50.4}\right)$ to the clinically negative neck region with a daily fraction size of 1.8 Gy and five fractions per week. RT was delivered using a sequential IMRT technique and a three‐phase treatment scheme: (a) 28 fractions containing all three PTVs, (b) five fractions containing ${\text{PTV}}_{59.4}$ and ${\mathrm{PTV}}_{72}$, and (c) seven fractions treated with ${\mathrm{PTV}}_{72}$ alone. The ${\mathrm{PTV}}_{72}$ plan was used to evaluate CI and HI (listed in Table 1).

**Table 1:** The dosimetric comparisons for DA‐VMAT and 7F‐IMRT.

*Parameter*	*Objective*	*7F‐IMRT*	*DA‐VMAT*	p‐*value*
${\text{PTV}}_{50.4}-V_{95\%}$	100%	99.13±0.18 (98.80–99.53)	99.01±0.32 (98.5–99.75)	n/s
${\text{PTV}}_{50.4}-V_{100\%}$	95%	95.09±0.34 (94.69–95.59)	95.13±0.23 (94.73–95.57)	n/s
${\text{PTV}}_{59.4}-V_{100\%}$	95%	95.37±0.80 (94.56–96.78)	95.12±0.21 (94.87–95.49)	n/s
${\text{PTV}}_{72}-V_{100\%}$	95%	95.54±0.39 (94.76–95.97)	95.41±0.27 (94.87–95.73)	n/s
${\text{PTV}}_{72}-V_{110\%}$	≤5%	2.15±1.07 (0.82–5.14)	1.70±0.83 (0–3.21)	n/s
CI	1	1.27±0.05 (1.21–1.37)	1.24±0.05 (1.16–1.31)	n/s
HI	1	1.11±0.02 (1.07–1.14)	1.09±0.02 (1.07–1.13)	<0.05

Abbreviations: IMRT: intensity‐modulated radiotherapy; 7F: 7‐field fixed‐beam IMRT; DA‐VMAT: dual arcs with the SmartArc technique; PTV: planning target volume; $V_{100\%}$: volume receiving ≥100% of the prescribed dose for the 95% of PTV; $V_{110\%}$: no more than 5% of any ${\mathrm{PTV}}_{72}$ will receive ≥110% of the prescribed dose; CI: conformity index; HI: homogeneity index; statistical significance (p<0.05) is reported from a paired two‐side Wilcoxon signed‐rank test; n/s: not statistically significant; numbers in fields were shown in the format of mean±standard deviation (range).

Based on the ICRU Report 62, the planning organ‐at‐risk volumes (PRVs) were defined as a safety margin around the organs at risk (OARs), particularly for a high‐dose gradient area. In this study, the PRV of the spinal cord was determined by adding a 3D margin of at least 5 mm to the delineated spinal cord. The PRVs of the brain stem and chiasm were defined through addition of a 3D margin of at least 1 mm around the delineated structures. According to the RTOG 0225,^(^ ^3^ ^,^ ^28^ ^)^ the planning objectives for PTVs were at a minimum dose >95%, and no more than 5% of any ${\mathrm{PTV}}_{72}$ received ≥110% of the prescribed dose. The structural constraints used in this study were as follows: 1) brain stem — maximum dose ≤54 Gy or 1% of PRV ≤60 Gy; 2) spinal cord — maximum dose ≤45 Gy or 1 cc of PRV ≤50 Gy; 3) chiasm — maximum dose ≤54 Gy or maximum dose of PRV ≤60 Gy; 4) parotid — mean dose ≤26 Gy or $V_{30\text{Gy}}\leq 50\%$; 5) eyes — maximum dose must be ≤45 Gy; 6) lens — maximum dose must be ≤10 Gy and as low as possible; 7) mandible — maximum dose must be ≤70 Gy or 1 cc of PRV and cannot exceed 75 Gy; 8) oral cavity excluding PTV — mean dose must be ≤40 Gy; and 9) healthy tissue — mean dose must be ≤30 Gy or no more than 1% or 1 cc of the tissue outside the PTV will receive ≥110% of the dose prescribed to the PTV.^(^ ^3^ ^)^

### E. 7F‐IMRT treatment planning study

The 7F‐IMRT plan was designed with a standard seven‐field coplanar arrangement. Plans were optimized with the DMPO option and generalized equivalent uniform dose (gEUD) module. The gEUD‐based optimization module was derived from the original definition of the equivalent uniform dose (EUD), based on a mechanistic formulation using a linear‐quadratic cell survival model.^(^ ^29^ ^,^ ^30^ ^)^ The DMPO option consisted of a fluence optimization with subsequent leaf sequencing for a few iterations (the sequencer creates a number of segments equal to or below the number predefined by the user). In such cases, the MLC position is considered in the optimization process and the resulting optimal fluence can be delivered by the linac without further approximations.^(^ ^14^ ^,^ ^31^ ^)^ The result of this first optimization is an initial guess of the segments. Subsequently, the leaf positions and weights are optimized with a gradient algorithm. The result is a set of MLC segments ready for delivery.^(^ ^32^ ^)^ Seven gantry angles of 60°, 100°, 140°, 180°, 220°, 260°, and 300° were set, with a maximum number of segments of 75. A weight factor, which defines the priority of each region of interest, must be assigned to each DVO. Having achieved these DVOs, the same dose volume constraints and weights were used for both VMAT and IMRT optimizations.

### F. Dual‐arc VMAT treatment planning (DA‐VMAT)

For ${\text{PTV}}_{50.4}$, DA‐VMATs were generated; all designed on the SmartArc TPS using a sequential method. In the DA‐VMAT planning, the arc was started with a gantry angle of 181° and was rotated clockwise for 358° to stop at the gantry angle of 179°. For the second arc, the gantry was rotated counterclockwise from 179° to 181°. Gantry rotation in clockwise and counterclockwise directions for the two arcs contributes to minimize the reset time. The two arcs shared the same isocenter, and no patient was repositioned between the first and second arc delivery. In the ${\text{Pinnacle}}^{3}$ SmartArc inverse planning module, the final dose was calculated at a gantry angle spacing of 2° with a total of 180 CPs per arc. The MLC leaf motion constraint was set to 5 mm for every 1° of gantry rotation. The collimator angle was set at 10° for all cases to minimize the cumulative effects of interleaf transmission and the tongue‐and‐groove effect.^(^ ^16^ ^,^ ^25^ ^,^ ^33^ ^)^ Regarding TPS dose rates, SmartArc allows seven different dose rate settings; however, according to the suggestions of Bedford and Warrington^(^ ^5^ ^)^ and Dobler et al.,^(^ ^23^ ^)^ we kept the five higher and dismissed the two lower dose rates to retain the main advantage in the faster delivery, when compared with IMRT. This also avoided not using the dose rates below 75 MU/min because of instabilities of the linac below 37 MU/min.^(^ ^5^ ^)^ Other user definable parameters were followed according to the literatures.^(^ ^17^ ^,^ ^24^ ^)^

For ${\text{PTV}}_{59.4}$ and ${\mathrm{PTV}}_{72}$, only a single arc is allowed by the SmartArc TPS when the sequential mode is running for VMAT. The first arc parameters in the dual arc were used for the plans. The dose distribution of ${\mathrm{PTV}}_{72}$ was used to evaluate the dose conformity and homogeneity in the target; for other parameters and OARs, all prescriptions will be cumulated and analyzed.

### G. Dose‐volume histogram (DVH) evaluation

Dose‐volume histogram plots were used to provide quantitative comparisons between the VMAT and IMRT treatment plans. The resolution of the dose calculation grid is 2.5 mm in the longitudinal direction and $4\times 4\;{\text{mm}}^{2}$ in the transversal plane for all IMRT and VMAT plans — thus making sure that they are unbiased for the subsequent computation of various indices. All data were based on the mean DVHs obtained from ${\text{Pinnacle}}^{3}$ with a bin size resolution of 0.01 Gy. Organ‐specific individual DVH values for all patients were calculated. The uncertainties of all values were reported as one standard deviation.

### H. Evaluation parameters

A detailed comparison between the VMAT and IMRT plans was evaluated using the following terms:

1. **Conformity index (CI):** $CI=V_{PTV}\times \frac{V_{TV}}{TV_{PV}^{2}}$, a ratio evaluating the coverage of the PTV to the prescription isodose volume in treatment plans, where $V_{TV}$ is the treatment volume of prescribed isodose lines, $V_{\mathrm{PTV}}$ is the volume of PTV, and ${\mathrm{TV}}_{\mathrm{PV}}$ is the volume of $V_{\mathrm{PTV}}$ within $V_{\mathrm{TV}}$. The smaller the value of CI, the better the conformal coverage.^(^ ^34^ ^,^ ^35^ ^)^
2. **Homogeneity index (HI):** $HI=\frac{D_{5\%}}{D_{95\%}}$, a ratio evaluating the dose homogeneity in PTV, where $D_{5\%}$ and $D_{95\%}$ are the minimum doses delivered to 5% and 95% of the PTV. A higher HI indicates poorer homogeneity.^(^ ^36^ ^,^ ^37^ ^)^
3. **Integral dose (DoseInt):** for healthy tissue, DoseInt (Gy·L) is defined as the integral of the absorbed dose extended over all voxels, excluding those within the target volume.
4. **Quality index (QI):** an index evaluating the difference in maximum or mean absorbed dose at serial or parallel OARs between different plans.^(^ ^36^ ^,^ ^38^ ^)^
  $$QI_{Serial}=D_{\max}^{DA-VMAT}/D_{\max}^{7F-IMRT},\;\text{and}\;\;\;\;QI_{Parallel}=D_{mean}^{DA-VMAT}/D_{mean}^{7F-IMRT}$$
5. **Comprehensive quality index (CQI):** the sum of each QI function times the weight of the OARs^(^ ^34^ ^,^ ^36^ ^,^ ^39^ ^)^ $(i.e.,\;CQI=\sum_{i=1}^{N}W_{i}\times {\left(QI_{Serial}|QI_{parallel}\right)}_{i}$ where *i* is the index of the critical structures and ‘|’ means ‘or’; $W_{i}$ denotes the priority weight for the OAR based on the demands of the physicians.) CQI values < 1 for an overall evaluation in the 11 OARs indicate a reduction in the maximal/mean dose for DA‐VMAT compared with 7F‐IMRT, and vice versa.
6. **EUD:** this module was derived by Niemierko and Emami et al.,^(^ ^40^ ^)^ who suggested a phenomenological model of the form. (Full details of which can be found in Refs. 40–42.)
  $$EUD={\left(\sum_{i=1}\left(v_{i}D_{i}^{a}\right)\right)}^{1/\alpha }$$
7. **Normal tissue complication probability (NTCP):** an EUD‐based NTCP model proposed by Niemierko^(^ ^40^ ^,^ ^41^ ^)^ was used to parameterize the dose‐response characteristics using the following logistic function:
  $$NTCP=\frac{1}{1+{\left(\frac{TD_{50}}{EUD}\right)}^{4_{\gamma 50}}}$$
  (Full details of which can be found in Ref. 41)

The values of a, $\gamma_{50}$, ${\mathrm{TD}}_{50}$, and α/β used in the present study are listed in Table 2 and are based on the models of Emami et al.,^(^ ^42^ ^)^ Lee et al.,^(^ ^43^ ^)^ Wu et al.,^(^ ^44^ ^)^ Taheri‐Kadkhoda et al.,^(^ ^45^ ^)^ and Scrimger et al.^(^ ^46^ ^)^ The Matlab‐2009a software (MathWorks, Inc., Natick, MA, USA) was used for EUD‐based NTCP calculations.

**Table 2:** Summary of the comparison results for the OARs.

*OAR\Endpoint (NTCP parameters)*	*Constraints*	*Parameter*	*7F‐IMRT*	*DA‐VMAT*	p‐*value*
Brain stem\necrosis (7, 3, 65, 3)	$D_{\max}\leq 54\text{Gy}$	$D_{\max}$	52.5±2.0 (49.9–55.7)	50.3±1.4 (47.7–51.9)	<0.05
		EUD	31.9±1.1 (29.7–32.7)	32.7±3.0 (27.1–35.6)	n/s
		NTCP	0.0±0.0 (0.0–0.0)	0.0±0.0 (0.0–0.0)	n/s
Spinal cord\myelitis (7.4, 4, 66.5, 3)	$D_{\max}\leq 45\text{Gy}$	$D_{\max}$	40.1±0.9 (38.8–41.6)	43.7±1.6(40.8–45.9)	<0.05
		EUD	21.9±2.4 (18.5–24.3)	23.9±3.8 (18.9–28.0)	n/s
		NTCP	0.0±0.0 (0.0–0.0)	0.0±0.0 (0.0–0.0)	n/s
Lt parotid gland\xerostomia (1, 2.2, 28.4, 8)	$D_{\mathrm{mean}}\leq 26\text{Gy}$	$D_{\mathrm{mean}}$	33.5±0.6 (32.6–34.8)	31.1±1.3 (28.9–34.0)	<0.05
	$V_{30}\leq 50\%$	$V_{30}$	39.1±3.5 (34.6–45.8)	36.6±3.4 (30.5–42.6)	<0.05
		EUD	27.2±0.5 (26.8–27.9)	25.9±1.0 (24.5–27.4)	<0.05
		NTCP	39.4±4.1 (33.2–45.7)	31.2±5.2 (24.0–40.0)	<0.05
Rt parotid gland\xerostomia (1, 2.2, 28.4, 8)	$D_{\mathrm{mean}}\leq 26\text{Gy}$	$D_{\mathrm{mean}}$	32.1±2.1 (28.8–37.4)	30.1±2.0 (27.3–34.1)	<0.05
	$V_{30}\leq 50\%$	$V_{30}$	41.8±3.1 (37.3–47.8)	34.6±2.9 (29.5–39.7)	<0.05
		EUD	26.0±2.5 (23.0–28.6)	25.5±2.4 (21.7–28.9)	n/s
		NTCP	33.1±8.2 (14.0–45.0)	31.3±8.3 (9.0–42.0)	n/s
Lt lens\cataract (3, 1, 18, 7)	$D_{\max}\leq 10\text{Gy}$	$D_{\max}$	8.0±2.9 (3.5–14.6)	7.6±2.6 (3.1–12.5)	n/s
		EUD	5.5±1.9 (2.2–7.5)	4.6±2.1 (1.8–6.9)	n/s
		NTCP	1.3±1.2 (0.04–2.7)	0.8±0.8 (0.01–1.9)	n/s
Rt lens\cataract (3, 1, 18, 7)	$D_{\max}\leq 10\text{Gy}$	$D_{\max}$	7.6±2.9 (3.8–12.4)	8.1±2.9 (3.4–14.5)	n/s
		EUD	5.3±1.8 (2.1–7.7)	5.1±2.5 (2.0–7.8)	n/s
		NTCP	1.1±1.0 (0.04–2.8)	1.3±1.3 (0.01–2.8)	n/s
Left side eye	$D_{\max}\leq 45\text{Gy}$	$D_{\max}$	24.7±7.4(16.7–40.7)	24.0±6.8 (15.2–37.8)	n/s
Right side eye	$D_{\max}\leq 45\text{Gy}$	$D_{\max}$	22.6±5.0 (15.0–32.6)	22.0±6.3 (12.8–34.2)	n/s
Chiasm	$D_{\max}\leq 54\text{Gy}$	$D_{\max}$	54.8±17.2 (25.5–71.4)	52.7±15.7 (32.0–70.1)	n/s
Mandible	$D_{1\text{cc}}\leq 75\text{Gy}$	$D_{1\text{cc}}$	68.6±5.6 (58.3–74.0)	66.7±4.6 (56.3–70.1)	n/s
Oral cavity	$D_{\mathrm{mean}}\leq 40\text{Gy}$	$D_{\mathrm{mean}}$	49.2±7.3 (40.4–60.3)	51.0±3.8 (43.2–58.3)	n/s
Healthy tissue	ALAP	$D_{\mathrm{mean}}$	33.3±3.1 (27.4–37.8)	31.7±4.0 (26.3–38.6)	n/s
		DoseInt	133.7±22.1 (102.9–159.8)	128.0±25.7 (97.1–159.6)	<0.05

Abbreviations: OAR: organ at risk; IMRT: intensity‐modulated radiotherapy; 7F‐IMRT: 7‐field fixed‐beam IMRT; DA‐VMAT: dual arcs with the SmartArc technique; Lt: left side; Rt: right side; Oral cavity: excluding planning target volume (PTV); DoseInt: Integral Dose [$10^{3}$ Gy·Litre]; EUD: equivalent uniform dose; NTCP: normal tissue complication probability (%), NTCP parameters used are listed in the parenthesis: $(a,\gamma_{50},{\text{TD}}_{50},\alpha /\beta );D_{\max}$; $D_{\max}$ : values were the maximal doses for a fixed volume fraction (V); $D_{\mathrm{mean}}$ : values were the mean doses for a fixed volume fraction (V); $V_{30}$: volume receiving 30 Gy for the 50% of parotid; n/s: not statistically significant; statistical significance (p<0.05) is reported from a paired two‐side Wilcoxon signed‐rank test; numbers in fields were shown in the format of mean±standard deviation (range); ALAP: as low as possible.

### I. Delivery evaluation

MU/fr, segments and delivery times for each plan were compared. The delivery time of all test runs for both plans was recorded by the same two experienced therapists. Patient setup time was not included.

Quality assurance (QA) was performed using an OCTAVIUS II phantom (PTW, Freiburg, Germany) made of polystyrene (physical density $1.04\;g/{\text{cm}}^{3}$, relative electron density 1.00; 32 cm wide and 32 cm long). A $30\times 30\times 2.2\;{\text{cm}}^{3}$ central cavity allows the user to insert the two‐dimensional (2D) ion chamber array into the phantom. To account for the couch attenuation, an 8 mm thick water‐equivalent contour was added under the phantom. The doses delivered were measured using the PTW 2D‐ARRAY seven29 (PTW, Freiburg, Germany) with a 2D detector matrix with 729 cubic ionization chambers.^(^ ^47^ ^)^ Comparison was performed using the Verisoft software (PTW), and gamma $\left(\Gamma_{2\;\text{mm},\;3\%}\right)$ evaluations^(^ ^15^ ^,^ ^16^ ^,^ ^48^ ^)^ were calculated (the dose criterion was ±3% and the distance criterion was 3 mm).

### J. Statistical analyses

Statistical tests of the differences between the DVH parameters of the IMRT and VMAT plans were performed using a two‐tailed Wilcoxon matched‐pair signed‐rank test (each pair in the test consisting of patient‐specific DVH values). Statistical significance was deemed a p‐value ≤0.05. SPSS software was used for data processing (ver. 16.0; SPSS, Inc., Chicago, IL, USA).

## III. RESULTS

**Figure 1:**
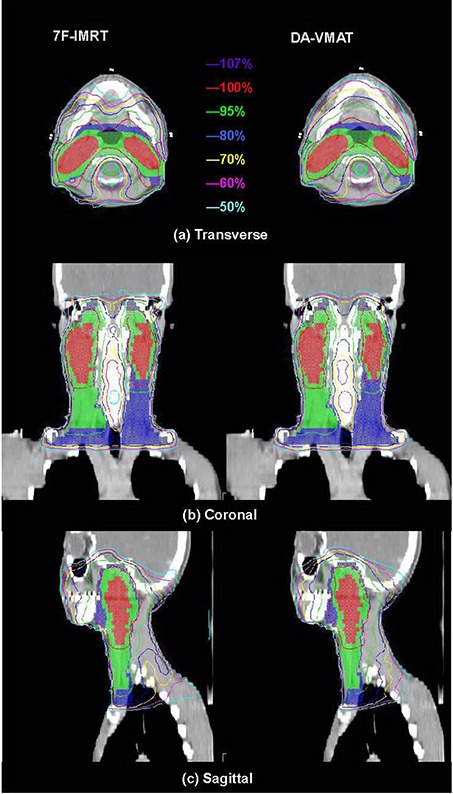
The isodose distributions on transverse, coronal, and sagittal views for one representative NPC case. Abbreviation: NPC: nasopharyngeal carcinoma

**Figure 2:**
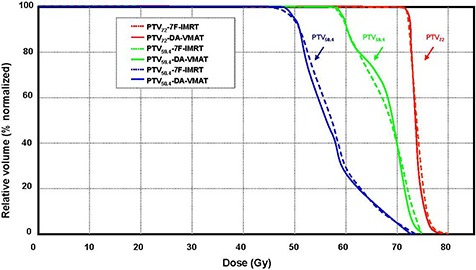
The mean DVHs for the PTV comparing DA‐VMAT (solid lines) to 7F‐IMRT (dotted lines). (Plan quality is slightly better for DA‐VMAT, with better target coverage and homogeneity. One outlier was not included in the mean DVH plot.) Abbreviations: DVH: dose volume histogram; PTV: planning target volume; DA‐VMAT: dual arcs with the SmartArc technique; 7F‐IMRT: 7‐field fixed beam intensity‐modulated radiotherapy

**Figure 3:**
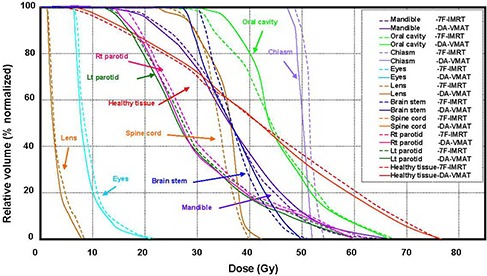
The mean DVHs for the selected OARs comparing DA‐VMAT (solid lines) to 7F‐IMRT (dotted lines). (Plan quality is slightly better for DA‐VMAT, with lower OAR doses. One outlier was not included in the mean DVH plot.) Abbreviations: DVH: dose volume histogram; DA‐VMAT: dual arcs with the SmartArc technique; 7F‐IMRT: 7‐field fixed beam intensity‐modulated radiotherapy; OAR: organ at risk

For all 18 patients included in this study, it was possible to achieve VMAT plans with dose distributions that would be acceptable for clinical treatment in our department if they had been produced by a standard IMRT plan. Thus, all 36 replans were approved by the same oncologist specializing in NPC. The isodose distributions on transverse, coronal, and sagittal views for one representative NPC case obtained using the 7F‐IMRT and DA‐VMAT are shown in Fig. 1. Tables 1 and 2 report the detailed numerical findings from the DVH analysis of PTV and OARs on all averaged cases, respectively. For the mean DVH plot, the single outlier was not included in Figs. 2 and 3.

### A. Target volume coverage, CI, and HI

As observed in Table 1, the two techniques fulfilled the objectives of ${\text{PTV}}_{50.4}-V_{95\%}$ and presented equally high target coverage (>99%), with no significant differences in the mean values observed in the paired test. For the objectives of ${\text{PTV}}_{50.4}{/}_{59.4}{/}_{72}-V_{100\%}>95\%$, similar results were obtained, with no significant differences in the mean values. Regarding the objectives of ${\text{PTV}}_{72}-V_{110\%}$, both techniques achieved the objective, and no significant difference was found between them. One outlier was observed from the DA‐VMAT with an outstanding ${\text{PTV}}_{72}-V_{110\%}$ value of zero.

Figure 2 shows the average DVHs of PTVs, comparing the two techniques for the entire cohort. Regarding the conformity of PTV, both techniques resulted in similar CI values (1.27±0.05 vs. 1.24±0.05 for 7F‐IMRT and DA‐VMAT, respectively). Regarding the homogeneity of PTV, we observed that the mean value of HI $\left(D_{5\%}/D_{95\%}\right)$ in DA‐VMAT was significantly better than that in 7F‐IMRT (1.09±0.02 vs. (1.11±0.02), indicating differences in the target homogeneity between the techniques.

### B. OAR sparing

The DVH analyses of specified OARs were as follows:

Brain stem: Planning objectives were met by both techniques. The mean values of $D_{\max}$ were significantly lower in DA‐VMAT. A dose reduction of $D_{\max}$ by (2.2±2.44) Gy was obtained from 7F‐IMRT to DA‐VMAT. In Fig. 3, an intersection occurred close to 36 Gy; however, the DA‐VMAT plans generated smaller mean $D_{\max}$ values.

Spinal cord: Both techniques also met the planning objective of $D_{\max}$ ≤45 Gy for the spinal cord. DA‐VMAT demonstrated poorer OAR sparing, with the mean values of $D_{\max}$ significantly lower in the 7F‐IMRT. A dose reduction of $D_{\max}$ by (3.6±1.84) Gy was obtained from DA‐VMAT to 7F‐IMRT.

Parotid glands: The planning objective was not fulfilled on the $D_{\mathrm{mean}}$ value for both parotids. However, a significant reduction in the mean dose of 2.2 Gy for the parotids was obtained by changing the treatment technique from 7F‐IMRT to DA‐VMAT (Table 2). Both techniques met the planning objective of $V_{30}\leq 50\%$. The mean DVHs for both parotid glands are shown in Fig. 3, indicating that the mean DVHs of DA‐VMAT were smaller.

Eyes: Planning objectives were met by both techniques, and no relevant differences were observed between the DA‐VMAT and 7F‐IMRT plans.

Chiasm and lenses: The DA‐VMAT plans yielded smaller $D_{\max}$ values for the chiasm and left lens, but not for the right one; however no significant dose differences were noted between the two techniques. The higher average $D_{\max}$ values for the chiasm occurred in the PRV area.

Mandible: No relevant differences were observed.

Oral cavity: The planning objective was not fulfilled on the $D_{\mathrm{mean}}$ value for the oral cavity. In contrast, a dose reduction of $D_{\mathrm{mean}}$ by (1.8±8.22) Gy was obtained from DA‐VMAT to7F‐IMRT, but this change was not statistically significant.

Healthy tissue: A statistically significant reduction in the DoseInt of 5.7 (Gy·L) was obtained by changing the treatment technique from 7F‐IMRT to DA‐VMAT.

Regarding tissue complications and NTCP values for specified OARs, only the left parotid gland (NTCP for xerostomia) was significantly lower in the DA‐VMAT plans versus the 7F‐IMRT plans (p<0.05).

Concerning CQI: values were < 1 in 11 OARs (0.98±0.02), indicating a reduction in the maximal/mean dose compared with 7F‐IMRT. The details of the QI and CQI values of the 11 selected OARs are listed in Table 3.

**Table 3:** The dosimetric comparisons of OARs for DA‐VMAT and 7F‐IMRT plans.

*Variables of OARs*	*Weight*	$QI_{7F}^{DA}$
*QI* of serial organs		
Brain stem	0.15	0.96±0.04
Spinal cord	0.15	1.03±0.03
Lt eye	0.075	0.98±0.14
Rt eye	0.075	0.98±0.08
Lt lens	0.075	0.99±0.10
Rt lens	0.075	1.02±0.17
Mandible	0.075	0.97±0.02
Chiasm	0.1	0.95±0.09
*QI* of parallel organs		
Lt parotid gland	0.075	0.97±0.04
Rt parotid gland	0.075	0.94±0.05
Oral cavity	0.075	1.05±0.10
*CQI* of eleven OARs		0.98±0.02

Abbreviations*:* IMRT: intensity‐modulated radiotherapy; 7F: 7‐field fixed‐beam IMRT; DA‐VMAT: dual arcs with the SmartArc technique; *QI*: quality index; Lt: left side; Rt: right side; *CQI*: comprehensive quality index; OAR: organ at risk; $QI_{7F}^{DA}$: *QI* of DA‐VMAT vs. 7F‐IMRT; numbers in fields were shown in the format of mean±standard deviation.

### C. Delivery efficiency and accuracy

The delivery efficiency and accuracy for the 18 patients are reported in Table 4. The MU/fr of 1.80 Gy resulted in an MU/fr reduction with DA‐VMAT of 21%, which was statistically significant. The mean segments used were lower with 7F‐IMRT.

**Table 4:** Delivery efficiency and accuracy for DA‐VMAT and 7F‐IMRT plans.

	*7F‐IMRT*	*DA‐VMAT*	p‐*value*
Segments	72.0±2.8 (69–75)	360	<0.05
MU/fr	602±57 (537–691)	474±48 (426–553)	<0.05
Delivery time (min)	8.7±0.9 (7.8–10.2)	4.6±0.7 (3.8–5.3)	<0.05
QA passing rate (%)	98.2±1.4 (95.0–99.8)	98.5±1.3 (95.3–99.9)	n/s

Abbreviations*:* IMRT: intensity‐modulated radiotherapy; 7F‐IMRT: 7‐field fixed‐beam IMRT; DA‐VMAT: dual arcs with the SmartArc technique; MU/fr: monitor units used per fraction; QA: quality assurance; n/s: not statistically significant; statistical significance (*p* <0.05) is reported from a paired two‐side Wilcoxon signed‐rank test; numbers in fields were shown in the format of mean±standard deviation (range).

Compared with 7F‐IMRT deliveries, DA‐VMAT deliveries were 47% faster, on average. Each technique yielded a high accuracy in dose delivery in terms of a high QA passing rate (>98%) of the $\Gamma_{3\;\text{mm},\;3\%}$ criterion.

## IV. DISCUSSION

Technologically, RT has progressed rapidly from 2D RT to 3D CRT, followed by IMRT, and currently VMAT. Advances in 3D CRT and IMRT have greatly improved both the physical conformity of treatment planning and RT delivery. IMRT delivers nonuniform dose distributions to the PTV, which represents significant progress. Generally, IMRT is a physically optimized form of RT. This is one solution for simultaneously treating the PTV while boosting as much of the GTV as possible without increasing the radiation dose to the critical OARs. Additionally, gantry arc‐based VMAT delivery methods have been proposed that involve the gantry rotating around the patient while MLC leaf positions, dose rate, and gantry speed are varied simultaneously during treatment, as developed by Otto.^(^ ^7^ ^)^ This was the precursor algorithm to Varian Medical Systems' RapidArc. SmartArc has recently become commercially available and can generate VMAT planning for both Elekta and Varian accelerators.^(^ ^16^ ^,^ ^17^ ^,^ ^24^ ^)^ VMAT offers the potential for reduced delivery times and improved dose distributions for difficult cases when compared with IMRT.^(^ ^6^ ^,^ ^7^ ^,^ ^15^ ^,^ ^16^ ^,^ ^25^ ^,^ ^30^ ^,^ ^49^ ^–^ ^50^ ^)^ However, VMAT has not been shown to provide general clinical advantages that can be translated to survival, tumor control, or normal tissue complications.

We report a comparison of VMAT by the SmartArc technique with dual coplanar arcs for NPC patients treated originally with a sequential step‐and‐shoot 7F‐IMRT technique. The performance investigation can be obtained through this comparison for a newly arrived TPS. To our knowledge, the comparison of SmartArc VMAT and IMRT in NPC using a sequential method has not been reported. The current study demonstrated that the DA‐VMAT achieved improved target coverage and showed improvement in sparing OARs and healthy tissue with respect to conventional 7F‐IMRT, while both delivery time and MU/fr decreased. The delivery time was equal to 4.6±0.7 min per dual arc and was considerably less than the 7F‐IMRT delivery time. Studies on other VMAT approaches based on double RapidArc approaches^(^ ^8^ ^,^ ^51^ ^,^ ^52^ ^)^ have shown delivery times ranging from 2.6 to 3.9 min that are faster than our results, whilst other improved IMAT approaches^(^ ^53^ ^,^ ^54^ ^)^ have displayed delivery times ranging from 6.3 to 14 min for conventional linac‐based techniques. Here, we did not determine the fastest method, as previous reports were not performed under the same measurement conditions. (Note: a faster RapidArc measurement (2.6 min) was performed by Clivio et al.^(^ ^8^ ^)^) Plans were optimized selecting a maximum dose rate of 600 MU/min with constant gantry speed rotating at the maximum speed allowed (5.5°/s) with a total of 177 CPs per arc, couch angle set to 0° and collimator set to either 30° or 36° (360°−X for the second arc), and managed all motions remotely in the reconfiguration state of the linac. VMAT plans created by the RapidArc algorithm can only be performed by the Varian machines. Moreover the beam data transfer time was not included.

The mean/maximal dose of the majority of the OARs was modestly reduced in the DA‐VMAT plans, with a mean value of CQI=0.98±0.02(DA‐VMAT vs. 7F−IMRT), indicating a 2% benefit in sparing OARs. This result shows that DA‐VMAT can deliver treatment in shorter times with an uncompromised or better target coverage and sparing of OARs, and that the SmartArc module in ${\text{Pinnacle}}^{3}$ serves as a robust VMAT planning tool capable of planning multiple arc deliveries for NPC cases. However, not all OARs — including the spinal cord and oral cavity — followed this sparing tendency, and the DA‐VMAT demonstrated worse OAR sparing. We could not determine the predominant reasons for this result, but it may have been caused by the single arc used on the ${\text{PTV}}_{59.4}$ and ${\mathrm{PTV}}_{72}$, which may reflect a drawback in the SmartArc TPS when the sequential mode is selected for VMAT.

It is worth noting that all plans in this study were clinically acceptable. Gamma analysis demonstrated that all plans were verified, with >95% of the measured points meeting the $\Gamma_{3\;\text{mm},\;3\%}$ criterion. However, not all 36 plans passed the $\Gamma_{3\;\text{mm},\;3\%}$ test at the first trial, and a replan was performed when the QA failed. One IMRT (T4N2M0) and a differing VMAT case (T4N2cM0) could not pass the >95% criterion. Based on the general experience of our clinic, no evidence indicated that the VMAT QA was more likely to pass compared with the IMRT or that failure would be observed in the same regions. However, the most likely phenomenon showed that the high gradient dose regions appeared to fail easily in the QA measurements. Further appropriate planning studies are therefore required for investigation into such complicated cases or for application in treating multiple targets within a large volume.^(^ ^16^ ^)^

Regarding tissue complications and NTCP values for the specified OARs, only the left side of parotid gland (NTCP for xerostomia) was significantly lower in the DA‐VMAT plans, indicating that a reduction in tissue complications may be achieved for the parotid glands. Different results in parotid sparing may relate to variations in the target volume definition, particularly for elective neck treatment. In this study, the higher prescribed doses and deep parotid lobes were always included in the PTVs, which inevitably lead to higher $D_{\mathrm{mean}}$ values in the majority of the glands. Kong et al.^(^ ^55^ ^)^ reported that the mean radiation dose to the right/left parotids was (35.6±5.3) Gy/(34.7±4.8) Gy, respectively, for sequential IMRT. Our data and other reports^(^ ^15^ ^,^ ^20^ ^,^ ^25^ ^)^ were similar. However, the DA‐VMAT plan significantly reduced the mean left parotid gland dose from 33.5 Gy (7F‐IMRT) to 31.1 Gy (DA‐VMAT) and the mean right parotid gland dose from 32.1 Gy to 30.1 Gy, respectively. We would expect a substantial gain in parotid gland sparing and target coverage if the 26 Gy is not a strict limitation.^(^ ^56^ ^)^ Additional reduction of xerostomia may be achieved by further sparing of the salivary glands and noninvolved oral cavity.

The main disadvantage of the VMAT planning technique compared with IMRT at present was the increased calculation time. The planning time was 12.7/47.7 min for ${\text{PTV}}_{50.4}$, the optimization time required was 9.6/14.2 min, and the dose calculation time required was 3.1/33.5 min on average in our experience for 7F‐IMRT / DA‐VMAT, respectively. The hardware used for the VMAT systems was as follows. For ${\text{Pinnacle}}^{3}$ SmartArc, a Philips 810X (64‐bit) Computer Workstation was used, as mentioned above. For RapidArc, a Dell Precision Workstation T5400 (32‐bit) Computer Workstation (CPU type: Intel Xeon Quad Core E5405/2GHz; 4GB RAM; Microsoft Windows XP operating system) was used. For Oncentra MasterPlan (v3.3 SP1; released in December 2009), an 8‐core processor (8 GB RAM, 64‐bit Windows system) was used. For Oncentra MasterPlan (v. 4.0; released in December 2010), a processor with 8 cores and a 64‐bit Windows system with 8 GB RAM and performing the dose calculation on a GPU processor of an NVIDIA Tesla C2505 graphics card was used. Calculation times could be reduced by future progress in computer science.

Debates continue about whether single or multiple arcs should be applied to obtain proper VMAT plan quality. Previous researchers showed that multiple arcs can improve both target coverage and the sparing of OARs.^(^ ^12^ ^,^ ^54^ ^,^ ^57^ ^,^ ^58^ ^)^ Recent studies^(^ ^18^ ^,^ ^25^ ^)^ on 65 HN cancer patients reported that multiple arcs were needed for such patients. For the more complex HN cancer cases, Shepard et al.^(^ ^18^ ^,^ ^19^ ^)^ concluded that the use of two arcs was necessary, leading to equivalent results as IMRT. Similar to other IMRT/VMAT planning reports, those found that tumors in HN regions, mainly NPC cases, were the most challenging cases. Vanetti et al. and Verbakel et al.^(^ ^12^ ^,^ ^13^ ^)^ showed that the double arc plans provided similar sparing of OARs and better PTV dose homogeneity than single arc or IMRT. This was one reason for excluding the single arc VMAT in the present study, and is why it is not considered an option in our clinical practice for NPC cases. Bertelsen et al.^(^ ^15^ ^)^ reported that the single arc had similar performance with IMRT in treating HN cancer cases, but was inconsistent with those that had more complex PTVs samples. However, the majority of publications state that two or more arcs are required.^(^ ^8^ ^,^ ^12^ ^,^ ^13^ ^,^ ^20^ ^,^ ^25^ ^,^ ^30^ ^)^ In our clinical experience, we not only observed that the difference of PTV coverage and OAR sparing existed, but also an impact of the dose calculation between single arc and dual arc VMAT occurred among the T3–4 cases whose lesions with the cranial border of the PTV became remarkable if the OARs were located on this level. A sharper PTV coverage in DA‐VMAT was observed when compared with single arc VMAT plans, and when the single arc was used, a calculation processing failure (stuck) may have occurred during optimization. Thus, this provides a second reason that the dual arc method was selected, smoothing the process to sculpt the complex shaping of the NPC cases. Bortfeld et al.^(^ ^6^ ^)^ additionally reported that, due to the complex target volumes and the frequent use of multiple prescription levels, HN cancer cases are most likely to experience significant dosimetric improvement when using more than one single arc VMAT.

For studies on RapidArc, Cozzi et al.^(^ ^9^ ^)^ and Fogliata et al.^(^ ^10^ ^)^ reported that the performance provided by the RapidArc to be consistent at least with similar target coverage, as well as sparing OARs in cervix uteri cancer and small benign brain tumors.^(^ ^8^ ^,^ ^11^ ^,^ ^59^ ^)^ Doornaert et al.^(^ ^20^ ^)^ showed that RapidArc achieved excellent target coverage and OARs sparing, with delivery completed in less than 3 min for HN cancer. For studies on MasterPlan, Alvarez‐Moret et al.^(^ ^21^ ^)^ showed that IMRT and double arc VMAT with similar result, but a single arc could not obtain a sufficient plan quality on HN cancers. Dobler et al.^(^ ^23^ ^)^ reported that single arc VMAT led to a similar plan quality compared with IMRT for prostate cancer. For treatment of hypopharynx/larynx cancer, a second arc was needed for obtaining adequate plan quality.

Our results are consistent with those reported in intercomparisons between double RapidArc $\left({\text{RA}}_{2}\right)$/MasterPlan dual arc and conventional IMRT in treating other sites of HN cancers.^(^ ^12^ ^,^ ^13^ ^,^ ^21^ ^,^ ^36^ ^,^ ^37^ ^,^ ^60^ ^)^ All of these studies found that double RapidArc $\left({\text{RA}}_{2}\right)$ provided equivalent or comparable planning results with conventional IMRT. Vanetti et al.^(^ ^12^ ^)^ observed that ${\mathrm{RA}}_{2}$ improved the homogeneity of dose distribution within PTV and PTV coverage together with a significantly greater sparing of OARs, compared with conventional IMRT and ${\mathrm{RA}}_{1}$ (single RapidArc).^(^ ^61^ ^)^

One limitation of running the sequential VMAT method in the SmartArc should be mentioned, namely that two independent trials cannot be merged with fluence maps to evaluate the real summation doses in the Pinnacle TPS. In the SmartArc planning, if a beam (static or step and shoot) is set to “None” for the optimization type and a dual arc optimization, is run, a fatal system error will occur at the conversion iteration. For ${\text{PTV}}_{59.4}$ and ${\mathrm{PTV}}_{72}$ planning, the optimization type was set to “None” for ${\text{PTV}}_{50.4}$ to account for all doses of PTVs, meaning only a single arc was permitted.

Additionally, we paid particular attention to reducing bias in the present study. Rather than using previously‐treated plans for reference, all the IMRT and VMAT plans were regenerated as new, using the same planning objectives. Having achieved these objectives, clinical experience at our center has demonstrated that more experienced IMRT planners produce superior dose distributions, so bias was minimized by cross‐planning with two equally experienced IMRT planners and with dose plans approved by an oncologist specializing in nasopharyngeal carcinoma (in our study the same oncologist reviewed all plans).

## V. CONCLUSIONS

Using SmartArc, DA‐VMAT produced plans with similar target coverage, as well as sparing OARs with 7F‐IMRT. The major difference between VMAT and IMRT for a sequential mode in treating NPC appears to be improved efficiency, resulting in a faster delivery time and the use of fewer MU/fr. A reduced effective delivery time may have a strong impact on clinical throughput, the individual management of patients, and the ability to perform systematic image guidance.

## ACKNOWLEDGMENTS

The authors thank the anonymous reviewers for their helpful comments on the original manuscript. This study was supported by grants from the NSC (100‐2221‐E‐151‐003) and CGMH (CMRPG890062).
